# Small molecule tyrosine kinase inhibitors approved for systemic therapy of advanced hepatocellular carcinoma: recent advances and future perspectives

**DOI:** 10.1007/s12672-024-01110-0

**Published:** 2024-07-03

**Authors:** Jianzhong Liu, Shuai Xia, Baoyi Zhang, Dina Mostafa Mohammed, Xiangliang Yang, Yanhong Zhu, Xinnong Jiang

**Affiliations:** 1 Clinical Laboratory, Wuhan No.7 Hospital, Zhong Nan 2nd Road, Wuhan, 430071 China; 2 Department of Biochemistry and Molecular Biology, Jining Medical University, Jining, 272067 Shandong China; 3 National Engineering Research Center for Nanomedicine, Key Laboratory of Molecular Biophysics of the Ministry of Education, College of Life Science and Technology, Huazhong University of Science and Technology, Wuhan, 430074 Hubei China; 4 Nutrition and Food Sciences Department, National Research Centre, Dokki, Cairo, Egypt

**Keywords:** Hepatocellular carcinoma, Tyrosine kinase inhibitor, Systemic therapy, Sorafenib, Lenvatinib, Donafenib, Regorafenib, Cabozantinib, Apatinib

## Abstract

Liver cancer is the sixth most commonly diagnosed cancer and the third leading cause of cancer death in the world, and hepatocellular carcinoma (HCC) is the most common form of liver cancer. More than half of the HCC patients are diagnosed at an advanced stage and often require systemic therapy. Dysregulation of the activity of receptor tyrosine kinases (RTKs) is involved in the development and progress of HCC, RTKs are therefore the potential targets for systemic therapy of advanced HCC (aHCC). Currently, a total of six small molecule tyrosine kinase inhibitors (TKIs) have been approved for aHCC, including first-line sorafenib, lenvatinib, and donafenib, and second-line regorafenib, cabozantinib, and apatinib. These TKIs improved patients survival, which are associated with disease stage, etiology, liver function, tumor burden, baseline levels of alpha-fetoprotein, and treatment history. This review focuses on the clinical outcomes of these TKIs in key clinical trials, retrospective and real-world studies and discusses the future perspectives of TKIs for aHCC, with an aim to provide up-to-date evidence for decision-making in the treatment of aHCC.

## Introduction

Liver cancer is the sixth most common cancer and the third leading cause of cancer death worldwide, with approximately 906,000 new cases and 830,000 deaths in 2020 [1]. In China, liver cancer is the fourth most commonly diagnosed cancer and the second leading cause of cancer death, with approximately 431,383 new cases and 412,216 deaths in 2022 [2]. The most common form of liver cancer is hepatocellular carcinoma (HCC) accounting for ~ 90% of cases [3]. HCC occurs most frequently in patients infected with hepatitis B virus (HBV) or hepatitis C virus (HCV), and approximately 43.0% and 19.0% of global HCC cases are caused by HBV and HCV infection respectively. Asian and African patients generally show a predominance of HBV infection, whereas American and most European patients show a prevalence of HCV infection [4, 5]. Most HCC patients have impaired liver function, the severity of which is assessed by Child–Pugh grade [6, 7] and albumin-bilirubin (ALBI) grade, with higher grade associated with worse liver function [8].

HCC is classified into 5 categories based on the Barcelona Clinic Liver Cancer (BCLC) staging system. Patients at stage 0 have a single tumor < 2 cm and Child–Pugh grade A with preserved liver function. Patients at stage A have either a single tumor or no more than 3 nodules (< 3 cm) and Child–Pugh grade A or B with partially impaired liver function. Patients at stage B have multinodular changes and Child–Pugh grade A–B. Patients at stage C have portal invasion and extrahepatic spread (EHS) and Child–Pugh grade A–B. Patients at stage D have Child–Pugh grade C with severely impaired liver function [9]. More than 50% of HCC patients are diagnosed at an advanced stage and 70% of patients relapse within the first 5 years of initial treatment. Early HCC is usually resectable, but advanced HCC (aHCC) often requires systemic therapy[10].

The pathophysiology of HCC is a multistep process associated with dysregulation of protein kinase activity [11]. Approximately 560 kinases have been identified in human kinome, among which 95 are tyrosine kinases that are divided into receptor tyrosine kinase (RTK) family with 58 members and nonreceptor tyrosine kinase (NRTK) family with 37 members [12, 13]. RTKs transduces extracellular signals (growth factors) to the cytoplasm, NRTKs relay intracellular signals. Ligand binding induces the dimerization, autophosphorylation, and activation of RTKs, leading to subsequent activation of multiple signaling cascades, such as Ras/Raf//MEK/ERK and PI3K/Akt pathways, which regulate cell proliferation, apoptosis, migration, and differentiation [14]. Abnormal activation of multiple RTKs, such as vascular endothelial growth factor receptors (VEGFRs), platelet-derived growth factor receptors (PDGFRs), epithelial growth factor receptors (EGFR), fibroblast growth factor receptors (FGFRs), and hepatocyte growth factor receptor (c-Met), have been detected in HCC [15].

**Fig. 1:**
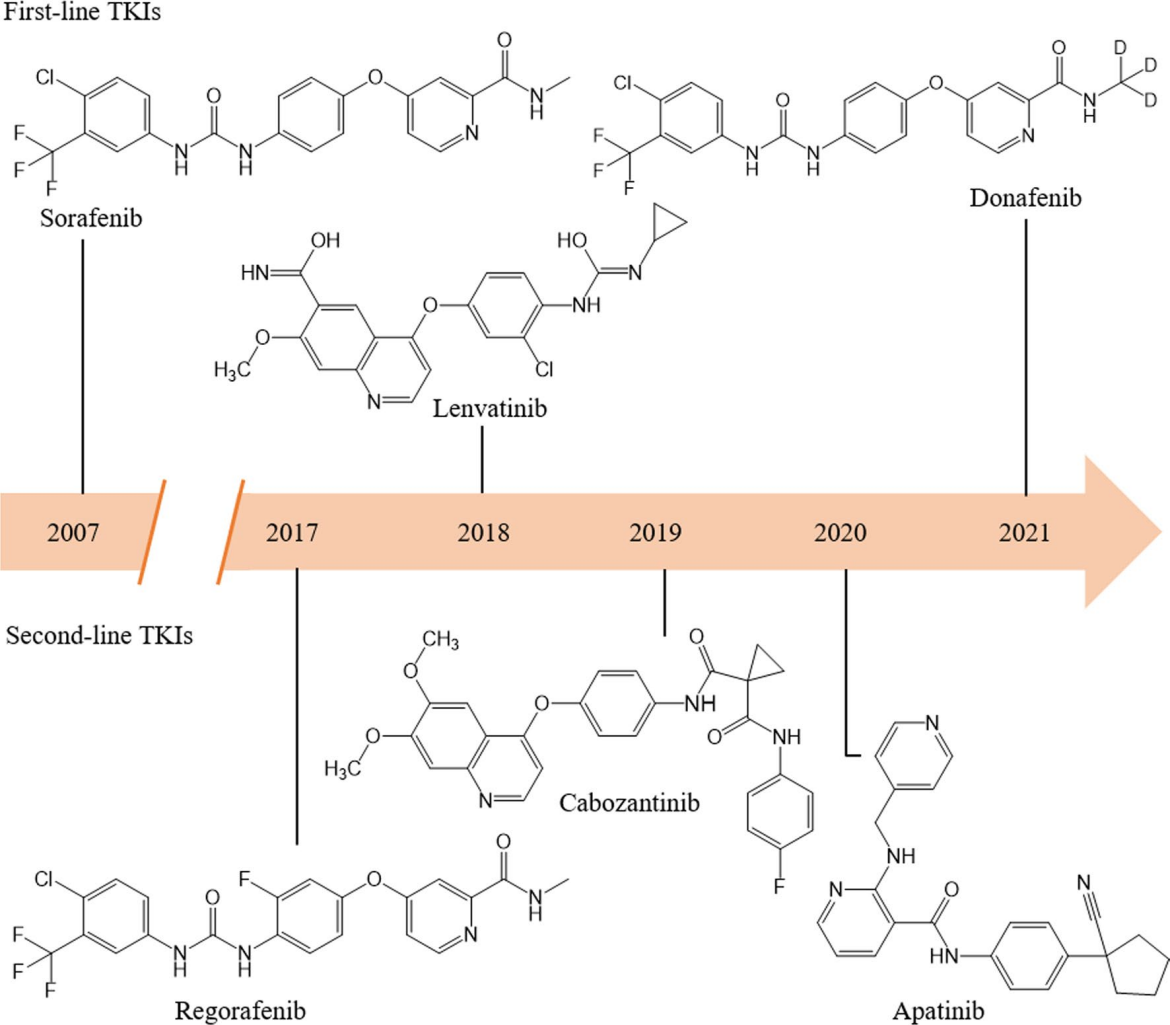
Molecular structures and timeline of small molecule tyrosine kinase inhibitors approved for advanced hepatocellular carcinoma**. **First-line sorafenib, lenvatinib, and donafenib are shown above the timeline, second-line regorafenib, cabozantinib, and apatinib are shown below the timeline. Sorafenib, lenvatinib, regorafenib, and cabozantinib were firstly approved by US Food and Drug Administration, donafenib and apatinib were firstly approved by National Medical Products Administration (NMPA) of China

Currently, a total of six small molecule inhibitors targeting RTKs have been approved for aHCC. Sorafenib, lenvatinib, and donafenib are first-line drugs, whereas regorafenib, cabozantinib, and apatinib are second-line drugs (Fig. 1). This review summarizes the efficacy and safety of these tyrosine kinase inhibitors (TKIs) in key clinical trials as well as in retrospective and real-world studies, compares the outcomes of lenvatinib versus (vs.) sorafenib, sorafenib vs. apatinib, and regorafenib vs. cabozantinib, and discusses the future perspective of TKIs for aHCC therapy.

## First-line therapy

### Sorafenib

**Fig. 2:**
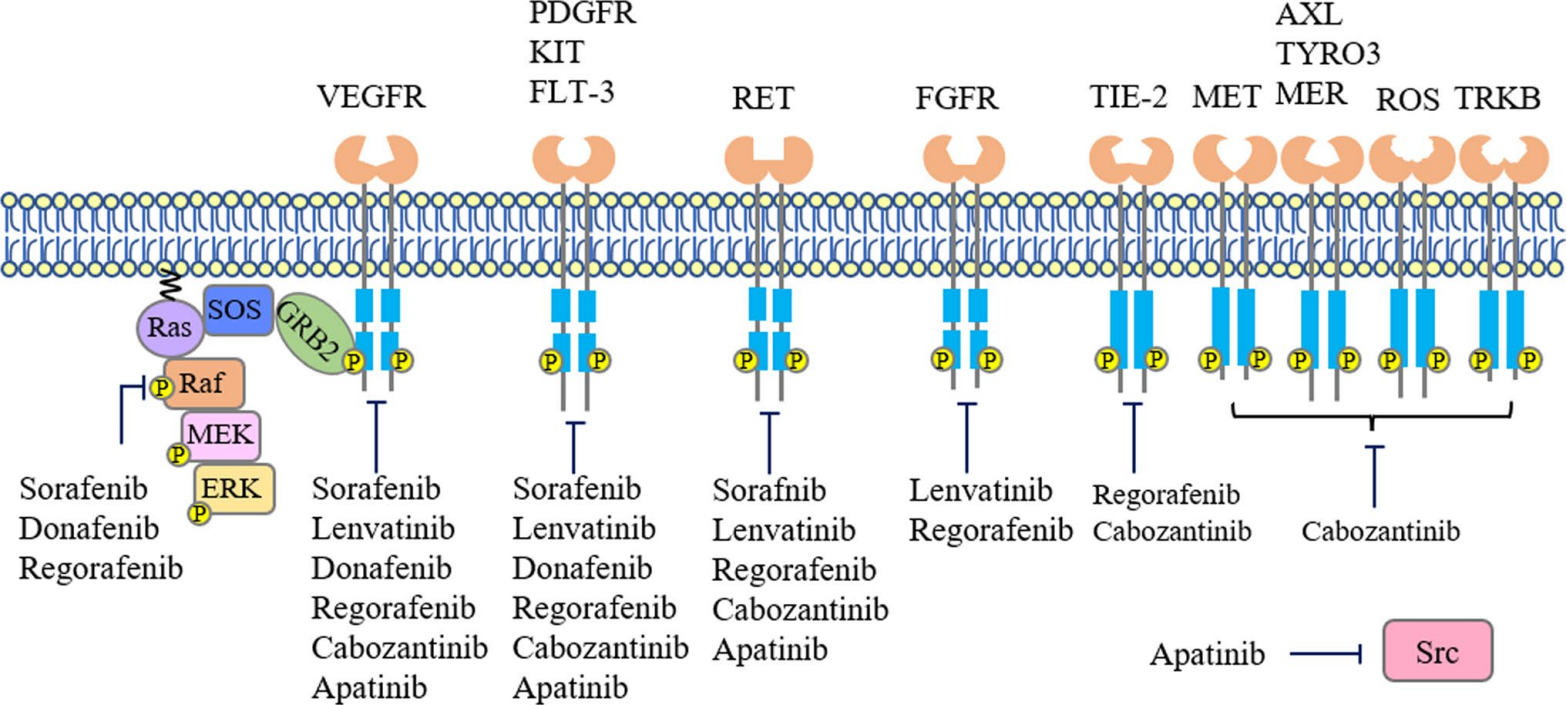
Receptor tyrosine kinases targeted by small molecule inhibitors approved for advanced hepatocellular carcinoma. All receptor tyrosine kinases (RTKs) have a similar molecular structure including extracellular ligand binding domains, a single transmembrane helix, cytoplasmic tyrosine kinase domain (TKD), and carboxy terminal region. Ligand binding induces dimerization, autophosphorylation, activation of RTKs and subsequent activation of multiple downstream signaling pathways. Raf and Src are nonreceptor tyrosine kinases. For simplicity, only Ras/Raf/MEK/ERK signaling cascade and one phosphorylation site on each kinase are shown in this figure. VEGFR, PDGFR, RET, and FGFR have split TKDs, which are indicated by two tandem blue frames

Sorafenib, a bi-aryl urea drug (Fig. 1) marketed by Bayer, Germany, was originally developed as a Raf1 (or C-Raf) kinase inhibitor [16]. Raf kinase isoforms, i.e., Raf1, A-Raf, and B-Raf, are the first tier of kinases in Ras/Raf/MEK/ERK signaling pathway [17]. Sorafenib inhibits the activities of Raf1, B-Raf, and oncogenic B-Raf^V600E^ by stabilizing the kinase domain in inactive conformation [18]. Sorafenib also potently inhibits several RTKs acting upstream of Raf, including angiogenic VEGFRs, PDGFR-β, FGFR-1, and tumorigenic Fms-like tyrosine kinase 3 (FLT-3), stem cell factor receptor (KIT), and rearranged during transfection (RET) [16] (Fig. 2).

#### Key clinical trials of sorafenib

In the randomized, placebo-controlled, phase 3 SHARP study (NCT00105443) conducted from March, 2005 to October, 2006 at 121 sites in 21 countries throughout Australia, Europe, North America, and South America, 602 patients with unresectable aHCC naive to systemic therapy were randomly assigned (1:1) to receive either 400 mg sorafenib or placebo twice daily in 6-week cycles. The median overall survival (OS) was 10.7 months for sorafenib and 7.9 months for placebo (P < 0.001) with a hazard ratio (HR) of 0.69 (95% CI 0.55–0.87; P < 0.001). The median time to progression (TPP) was 5.5 months for sorafenib and 2.8 months for placebo (HR: 0.58; 95% CI 0.45–0.74; P < 0.001). According to Response Evaluation Criteria in Solid Tumors (RECIST), complete response (CR), partial response (PR), and stable disease (SD) were 0%, 2%, and 71% for sorafenib, and 0%, 1%, and 67% for placebo, respectively. Sorafenib had a significantly higher disease control rate (DCR; CR plus PR plus SD) than placebo (43% vs. 32%; P = 0.002) [19] (Table 1).

**Table 1:** Key clinical trials of small molecule receptor tyrosine kinase inhibitors approved for systemic therapy of advanced hepatocellular carcinoma

RTK inhibitor	Manufacturer	Targets	Study (trial identifer)	Study arm (patient No.)	Setting	mOS (months)	mPFS (months)	mTTP (months)	ORR	DCR	Approved years	References
Sorafenib (BAY-43–9006; Nexavar)	Bayer	RAF-1, VEGFR1-3, PDGFR-β, FGFR1, FLT-3, KIT, RET	SHARP (NCT00105443)	Sorafenib (299) Placebo (303)	First-line	10.7 vs. 7.9 (HR: 0.69; 95% CI 0.55–0.87; P < 0.001)	–	5.5 vs. 2.8 (HR: 0.58; 95% CI 0.45–0.74; P < 0.001)	2% vs. 1% (P = 0.05)	43% vs. 32% (P = 0.002)	2007, US	[16, 19]
			Asia–Pacific (NCT00492752)	Sorafenib (176) Placebo (76)		6.5 vs. 4.2 (HR: 0.68; 95% CI 0.50–0.93; P = 0.014)	–	2.8 vs. 1.4 (HR: 0.57; 95% CI 0.42–0.79; P = 0.0005)	3.3% vs. 1.3%	35.3% vs. 15.8% (P = 0.0019)		[20]
Lenvatinib (E7080; Lenvima)	Eisai Inc	VEGFR1-3, FGFR1-4, PDGFR-α, RET, KIT	REFLECT (NCT01761266)	Lenvatinib (478) Sorafenib (476)	First-line	13.6 vs. 12.3 (HR: 0.92; 95% CI 0·79–1·06)	7.4 vs. 3.7 (HR: 0·66; 95% CI 0·57–0·77; P < 0.0001)	8.9 vs. 3.7 (HR: 0.63; 95% CI 0.53–0.73; P < 0.0001)	24.1% vs. 9.2% (OR: 3·13; 95% CI 2·15–4·56; P < 0.0001)	75.5% vs. 60.5%	2018, US	[38, 41]
Donafenib (CM4307; Zepsun)	Suzhou Zelgen Biopharm	RAF-1, VEGFR1-3, PDGFR-β, FGFR1, FLT-3, KIT, RET	ZGDH3 (NCT02645981)	Donafenib (328) Sorafenib (331)	First-line	12.1 vs. 10.3 (HR: 0.83; (95% CI 0.70–0.99; P = 0.0245)	3.7 vs. 3.6 (HR: 0.91; 95% CI 0.76–1.09; P = 0.0570)	3.7 vs. 3.7 (HR: 0.931; 95% CI 0.78–1.12; P = 0.1029)	4.6% vs. 2.7%; (P = 0.2448)	30.8% vs. 28.7% (P = 0.5532)	2021, China	[54, 55]
Regorafenib (BAY 73–4506; Stivarga)	Bayer	RET, KIT, BRAF, RAF-1, BRAF^V600E^, VEGFR1-3, FGFR1, PDGFR-β, TIE-2	RESORCE (NCT01774344)	Regorafenib (379) Placebo (179)	Second-/Third-line	10.6 vs. 7.8 (HR: 0.63; 95% CI 0.50–0.79; P < 0.0001)	3.1 vs. 1.5 (HR: 0.46; 95% CI 0.37–0.56; P < 0.0001)	3.2 vs. 1.5 (HR: 0.44; 95% CI 0.36–0.55; P < 0.0001)	11% vs. 4% (P = 0.0047)	65% vs. 36% (P < 0.0001)	2017, US	[57–59]
Cabozantinib (XL184; Cabometyx/Cometriq)	Exelixis Inc	VEGFR1-3, MET, ROS1, RET, AXL, NTRK, KIT	CELESTIAL (NCT01908426)	Cabozantinib (470) Placebo (237)	Second-line	10.2 vs. 8.0 (HR: 0.76; 95% CI 0.63–0.92; P = 0.005)	5.2 vs. 1.9 (HR: 0.44; 95% CI 0.36–0.52; P < 0.001)	–	4% vs. 0.4% (P = 0.009)	64% vs. 33% (P < 0.001)	2019, US	[70, 71]
Apatinib (YN968D1; Aitan)	Jiangsu Hengrui Medicine	VEGFR-2, KIT, RET, c-Src	AHELP (NCT02329860)	Apatinib (267) Placebo (133)	Second-line	8.7 vs. 6.8 (HR: 0.79; [95% CI 0·62–1.00; P = 0.048)	4.5 vs. 1.9 (HR: 0·47; 95% CI 0·37–0·60; P < 0.0001)	4.7 vs. 1.9 (HR: 0·43; [95% CI 0·33–0·57; P < 0.0001)	11% vs. 2%	61% vs. 29%	2020, China	[84, 86]

*CI* confidence interval, *DCR* disease control rate, *HR* hazard ratio, *mPFS* median progress-free survival, *mOS* median overall survival, *mTTP* median time to progress, *ORR* objective response rate, *OR* odds ratio

In the randomized, double-blinded, placebo-controlled, phase 3 Asia–Pacific study (NCT00492752) conducted between September, 2005 and January, 2007 at 23 centers in China, South Korea, and Taiwan, 226 patients were randomized (2:1) to sorafenib or placebo. Sorafenib vs. placebo remarkably extended median OS (6.5 vs. 4.2 months; HR: 0.68; 95% CI 0.50–0.93; P = 0.014) and TTP (2.8 vs. 1.4 months; HR: 0.57; 95% CI 0.42–0.79; P = 0.0005). CR, PR, SD, and progressive disease (PD) were 0%, 3.3%, 54%, and 30.7% for sorafenib, and 0%, 1.3%, 27.6%, and 54.0% for placebo, respectively. Accordingly, DCR was significantly higher for sorafenib than for placebo (35.3% vs. 15.8%; P = 0.0019) [20] (Table 1).

The overall incidence of treatment-related adverse events (AEs) was 80% for sorafenib vs. 52% for placebo in SHARP study, and 81.9% for sorafenib vs. 38.7% for placebo in Asia–Pacific study. The most common drug related AEs were hand-foot skin reaction (HFSR), diarrhea, alopecia, anorexia, fatigue, and hypertension, which were well-tolerated and manageable [19, 20].

Sub-analyses of SHARP and Asia–Pacific studies showed that HCV-positive patients in the sorafenib group had significantly improved TTP (HR: 0.39; 95% CI 0.25–0.62; P < 0.0001) and OS (HR: 0.52; 95% CI 0.36–0.76; P < 0.0006) vs. those in the placebo group, whereas HBV-positive patients showed a trend in favor of sorafenib rather than placebo for TTP (HR: 0.74; 95% CI 0.48–1.14; P = 0.18) and OS (HR: 0.74; 95% CI 0.544–1.03; P = 0.08) [21]. Sorafenib was therefore beneficical to HBV- and HCV-positive patients in terms of TTP and OS.

The US Food and Drug Administration (FDA) approved sorafenib in 2007 as a first-line therapy for aHCC based on SHARP and Asia–Pacific studies, which demonstrated that sorafenib was safe and significantly improved median OS, TTP, and DCR compared with placebo. In the single-arm, open-label, phase 4 HATT study (NCT01098760) conducted from August, 2010 to October, 2013 across 7 sites in Taiwan, sorafenib achieved a median OS, progression-free survival (PFS), and TTP of 8.6, 2.7, and 3.8 months respectively in aHCC patients (n = 151). No patients achieved CR, objective response rate (ORR; CR plus PR) was 6.6% and DCR was 47.7%. The most frequent grade ≥ 3 drug-related AEs were HFSR, diarrhea, and hypertension [22]. These results were comparable to those observed in SHARP and Asia–Pacific studies [19, 20].

#### The outcomes of sorafenib in retrospective studies

In aHCC patients with various organ lesions (n = 377), sorafenib had a median OS, ORR, and DCR of 11.4 months, 19.4%, and 54.7% respectively. Patients with bone metastasis, lung metastasis, macrovascular invasion (MVI) or multiple lesions had significantly worse PFS compared with those without corresponding lesion(s) (P = 0.021, 0.036, 0.038, and 0.009, respectively) [23]. Compared with patients enrolled in SHARP [19], Asia–Pacific [20], and HATT [22] trials, patients in this study had numerically better OS and tumor responses.

In SHARP and Asia–Pacific studies, CR to sorafenib was not achieved and ORR was low (≤ 3.3%) according to RECIST [19, 20]. In aHCC patients enrolled in phase 3 SILIUS study (NCT01214343), response to sorafenib was assessed according to modified RECIST (mRECIST), which revealed an ORR of 18.8%. Sorafenib responders (CR plus PR) had a significantly higher median OS than non-responders (SD plus PD) (27.2 vs. 8.9 months; P < 0.0001) [24, 25]. Analysis of 12 retrospective studies (including 911 aHCC patients) that evaluated response to sorafenib according to mRECIST revealed a pooled ORR of 16.9% (95% CI 9.9–23.9) and pooled median OS of 12.2 months (95% CI 10.7–13.8). CR was achieved in 5.8% (95% CI 1.1–10.5) patients with a median OS of 24.0 months (95% CI 14.6–33.4), PR was achieved in 11.4% (95% CI 5.5–17.2) patients with a median OS of 20.1 months (95% CI 15.9–25.4), SD was observed in 45.7% (95% CI 39.5–521.9) patients with a median OS of 11.7 months (95% CI 9.7–13.6), and PD was observed in 33.0% (95% CI 24.2–41.8) patients with a median OS of 7.7 months (95% CI 6.6–8.8). Responders had remarkably longer median OS vs. non-responders (CR vs. SD: P = 0.0018; CR vs. PD: P = 0.0002; PR vs. SD: P = 0.0003; PR vs. PD: P < 0.0001). In addition, median OS was notably better in patients with SD than in those with PD (P = 0.0010) [26–37] (Table 2). mRECIST can therefore identify more sorafenib responders than RECIST, and responders tend to have better median OS than non-responders.

**Table 2:** The efficacy of sorafenib in retrospective studies

Reference	Patient number	mOS (months)	ORR (%)	CR		PR		SD		PD	
				N (%)	mOS	N (%)	mOS	N (%)	mOS	N (%)	mOS
Arizumi et al. [26]	158	15.6	22.8	6 (3.8)	25.4	30 (19.0)	25.4	52 (32.9)	6.3	70 (44.3)	7.3
Edeline et al. [27]	53	9.7	22.6	2 (3.8)	18.2	10 (18.9)	18.2	30 (56.6)	9.7	11 (20.8)	6
Fukubayashi et al. [28]	72	12.5	15.3	3 (4.2)	20	8 (11.1)	20	43 (59.7)	14.4	18 (25.0)	8.9
Gavanier et al. [29]	60	10.5	6.7	0 (0)	–	4 (6.7)	17.5	27 (45.0)	9.5	29 (48.3)	9.1
Hatooka et al. [30]	48	15	6	1 (2.1)	–	2 (4.2)	33	29 (60.4)	18	13 (27.1)	9
Kawaoka et al. [31]	41	10	4.8	1 (2.4)	NR	1 (2.4)	NR	18 (43.9)	10	17 (41.5)	7
Kawaoka et al. [32]	66	8.6	9.1	2 (3.0)	NR	4 (6.1)	NR	26 (39.4)	10	17 (25.8)	5
Moschouris et al. [33]	21	–	38.1	2 (9.5)	21.5	6 (28.6)	21.5	11 (52.4)	12.2	2 (9.5)	–
Ocal et al. [34]	115	14.3	29.5	6 (5.2)	49.1	28 (24.3)	17.6	50 (43.5)	14.3	31 (27.0)	8
Ronot et al. [35]	64	12.8	28.1	18 (28.1)	25.5	0 (0)	–	29 (45.3)	13.3	17 (26.6)	5.7
Takada et al. [36]	191	13.1	10.8	5 (2.6)	22	20 (10.5)	22	72 (37.7)	10	78 (40.8)	10
Yamamichi et al. [37]	22	12.6	9.1	1 (4.5)	10.4	1 (4.5)	10.4	7 (31.8)	12.6	13 (59.1)	9
Means (95% CI)		12.2 (10.7–13.8)	16.9 (9.9–23.9)	5.8 (1.1–10.5)	24.0 (14.6–33.4)	11.4 (5.5–17.2)	20.1^a^ (15.9–25.4)	45.7 (39.5–51.9)	11.7^b,c^ (9.7–13.6)	33.0 (24.2–41.8)	7.7^d,e,f^ (6.6–8.8)
Means ± SD		12.2 ± 2.3	16.9 ± 11.0	5.8 ± 7.4	24.0 ± 11.2	11.4 ± 9.2	20.1 ± 6.2	45.7 ± 9.7	11.7 ± 3.1	33.0 ± 13.8	7.7 ± 1.6

Unpaired* t* test was performed to analyze mOS, CR, PR, SD, and PD

*CR* complete response, *mOS* median overall survival, *NR* not reached, *ORR* objective response rate, *PR* partial response, *PD* progressive disease, *SD* stable disease

^a^CR vs. PR, P = 0.4451

^b^CR  vs. SD, P = 0.0018

^c^CR  vs. PD, P = 0.0002

^d^PR vs. SD, P = 0.0003

^e^PR vs. PD, P < 0.0001

^f^SD  vs. PD, P = 0.0010. P < 0.05 were considered to be significantly different

Sorafenib remained the only first-line systemic therapy for aHCC patients for 10 years until the approval of lenvatinib.

### Lenvatinib

Lenvatinib, a urea derivative (Fig. 1) developed by Eisai Inc., USA, is a multi-kinase inhibitor of VEGFRs, FGFRs, PDGFR-α, RET, and KIT [38] (Fig. 2).

#### Key clinical trials of lenvatinib

In a single arm, phase 2 clinical trial (NCT00946153), aHCC patients naïve to systemic therapy (n = 46) received lenvatinib (12 mg daily) in 28-day cycles. Lenvatinib had a median OS, median TTP, and ORR of 18.7 months, 7.4 months, and 37%, respectively, and lower body weight (BW) was associated with earlier drug withdrawal or dose reduction due to treatment-emergent AEs [39]. A BW-based lenvatinib daily dosing was thereby reccommended for HCC patients, i.e., 12 mg and 8 mg for BW ≥ 60 kg and < 60 kg respectively [40].

In the multicenter, randomized, open-labelled, phase 3 REFLECT study (NCT01761266) conducted between March, 2013 and July, 2015 at 154 sites in 20 countries across the Asia–Pacific, European, and North American regions, 954 patients with unresectable aHCC were randomized (1:1) to lenvatinib or sorafenib with BW-based lenvatinib dosing. Lenvatinib was non-inferior to sorafenib in terms of median OS (13.6 vs. 12.3 months; HR: 0.92; 95% CI 0.79–1.07; P > 0.05) and remarkably improved median PFS (7.4 vs. 3.7 months; HR: 0.66; 95% CI 0.57–0.77; P < 0.0001) and median TTP (8.9 vs. 3.7 months; HR: 0.63; 95% CI 0.53–0.73; P < 0.0001) compared with sorafenib. CR, PR, SD, and PD were 1%, 23%, 51%, and 15% for lenvatinib, and < 1%, 9%, 51%, and 31% for sorafenib, respectively. Accordingly, lenvatinib had a significantly higher ORR [24.1% vs. 9.2%; odds ratio (OR): 3.1; 95% CI 2.15–4.56; P < 0.0001] and numerically better DCR (75.5% vs. 60.5%) than sorafenib (Table 1). The most common AEs were hypertension, diarrhea, decreased appetite, and decreased weight for lenvatinib, and palmar-plantar erythrodysaesthesia (PPE), diarrhea, hypertension, and decreased appetite for sorafenib. Lenvatinib showed a higher probability of grade 3–5 AEs than sorafenib with an OR of 1.51 (95% CI 1.14–2.00) [41, 42]. FDA approved lenvatinib as a first-line therapy for aHCC in 2018 based on the results of REFLECT study.

Exploratory analysis of REFLECT data revealed that patients in higher or lower BW groups had similar median OS (13.7 vs. 13.4 months) and the same PFS (7.4 months). Treatment-emergent AEs required dose modifications in 57.5% and 43.0% of patients in the higher and lower BW groups respectively [43]. The similar efficacy and safety results between the two BW groups support the BW-based lenvatinib dosing for patients with aHCC.

Of the HBV-positive patients in REFLECT study, median OS was 13.4 months for lenvatinib and 10.2 months for sorafenib (HR: 0.83; 95% CI 0.68–1.02). Median PFS was 7.3 months for lenvatinib and 3.6 months for sorafenib (HR: 0.62; 95% CI 0.50–0.75) [41]. Casadei Gardini et al. revealed a clear trend in favor of lenvatinib over sorafenib (HR 0.82; 95% CI 0.60–1.15) in terms of OS in HBV-positive patients [21]. Choi et al. reported that HBV-positive patients on lenvatinib vs. those on sorafenib had comparable OS (7.0 vs. 9.2 months; P = 0.070) and PFS (4.6 vs. 2.4 months; P = 0.134), significantly longer TTP (5.2 vs. 2.5 months; P = 0.018), and notably higher ORR (18.2% vs. 4.5%; P = 0.001) and DCR (77.3% vs. 47.7%, P = 0.001) [44]. These data suggested that HBV-positive patients may benefit more from lenvatinib than from sorafenib.

#### The outcomes of lenvatinib in real-world and retrospective studies

In REFLECT study, the enrolled patients had not received systemic treatment previously and were categorized to BCLC stages B or C and Child–Pugh grade A; those with ≥ 50% liver occupation, main branch portal vain thrombosis (PVT), and bile duct invasion (BDI) were excluded (referred to as REFLECT criteria) [41]. However, aHCC patients may not meet the REFLECT criteria (REFLECT-ex) in real-world setting. The outcomes of lenvatinib in REFLECT-in (meet the REFLECT criteria) vs. REFLECT-ex patients were evaluated in real-world and retrospective studies.

Wang et al. systemically reviewed 18 studies including both REFLECT-in and REFLECT-ex patients (n = 1719) and found that lenvatinib had a pooled median OS, median PFS, ORR, and DCR of 10.80 months (95% CI 7.17–14.43), 6.44 months (95% CI 5.01–7.86), 36% (95% CI 26.0–45.0%), and 74% (95% CI 65.0–83.0%) respectively [45]. REFLECT-in patients had numerically better median OS (18.3 vs. 11.4 months; P = 0.1643) [45–47], median PFS (8.8 vs. 6.7 months; P = 0.1834) [45–48], ORR (42.2% vs. 37.0%; P = 0.7416), and DCR (81.5% vs. 75.8%; P = 0.6559) [46–49] compared with REFLECT-ex patients (Table 3). The rate of grade > 3 AEs was similar for REFLECT-in and REFLECT-ex patients [48]. Hence, lenvatinib had comparable efficacy and safety profile in REFLECT-in and REFLECT-ex patients.

**Table 3:** The outcomes of lenvatinib in REF-in and REF-ex patients

References	Patient No				mOS (months)		mPFS (months)		ORR (%)		DCR (%)	
	REF-in	REF-ex	CPA	CPB	REF-in vs. REF-ex	CPA vs. CPB	REF-in vs. REF-ex	CPA vs. CPB	REF-in vs. REF-ex	CPA vs. CPB	REF-in vs. REF-ex	CPA vs. CPB
Cheon et al. [50]	57	35	74	18	–	7.4 vs. 5.3 (P = 0.039)	–	4.4 v.s 2.6 (P = 0.377)	–	16.2 vs. 5.6 (P = 0.451)	–	83.8 vs. 61.1
Choi et al. [44]	–	–	29	15	–	10.8 vs. 5.0	–	4.6 vs. 3.7	–	–	–	–
Goh et al. [46]	48	63	–	–	13.9 vs. 8.7 (P = 0.006)	–	6.9 vs. 5.2 (P = 0.10)		20.8 vs. 17.5 (P = 0.81)	–	85.4 vs. 68.3 (P = 0.045)	–
Maruta et al. [51]	–	–	132	20	–	13.3 vs. 8.1 (P = 0.167)	–	5.1 vs. 4.3 (P = 0.795)	–	39 vs. 50	–	60 vs. 65
Singal et al. [52]	–	–	104	91	–	–	–	–	–	58.7 vs. 70.3	–	86.6 vs. 96.7
Sho et al. [49]	18	23	–	–	–	–	–	–	61.1 vs. 60.9 (P = 0.8293)	–	88.9 vs. 91.3 (P = 0.7965)	–
Sho et al. [48]	41	64	75	24	–	–	10.3 vs. 9.8 (P = 0.78)	10.9 vs. 8.6 (P = 0.25)	61.5 vs. 48.3 (P = 0.1967)	56.0 vs. 45.8 (P = 0.3852)	87.2 vs. 95.0 (P = 0.1688)	90.7 vs. 95.8
Tsuchiya et al. [45]	174	71	276	67	25.3 vs. 15.2	21.0 vs. 9.0	10.0 vs. 7.1	8.8 vs. 5.1	–	–	–	–
Welland et al. [47]	110	95	160	45	15.6 vs. 10.2 (P = 0.002)	13.8 vs. 6.1 (P < 0.001)	8.1 vs. 4.8 (P = 0.011)	–	25.5 vs. 21.1 (P = 0.729)	–	64.5 vs. 48.4 (P = 0.063)	–
Means (95% CI)					18.3 (3.0–33.5) vs. 11.4 (2.9–19.8) (P = 0.1643)	13.3 (7.0–19.5) vs. 6.7 (4.5–8.9) (P = 0.0247)	8.8 (6.3–11.4) vs. 6.7 (3.1–10.4) (P = 0.1834)	6.8 (3.1–10.4) vs. 4.9 (2.0–7.7) (P = 0.2854)	42.2 (7.0–77.4) vs. 37.0 (3.4–70.5) (P = 0.7416)	42.5 (11.3–73.6) vs. 42.9 (− 0.2–86.0) (P = 0.9794)	81.5 (63.3–99.7) vs. 75.8 (41.2–110.3) (P = 0.6559)	80.3 (58.3–102.3) vs. 79.7 (49.0–110.3) (P = 0.9596)
Means ± SD					(18.3 ± 6.2) vs. (11.4 ± 3.4)	(13.3 ± 5.0) vs. (6.7 ± 1.8)	(8.8 ± 1.6) vs. (6.7 ± 2.3)	(6.8 ± 2.9) vs. (4.9 ± 2.3)	(42.2 ± 22.1) vs. (37.0 ± 21.1)	(42.5 ± 19.6) vs. (42.9 ± 27.1)	(81.5 ± 11.4) vs. (75.8 ± 21.7)	(80.3 ± 13.8) vs. (79.7 ± 19.2)

*CPA* Child–Pugh grade A, *CPB* Child–Pugh grade B, *DCR* disease control rate, *mPFS* median progression-free survival, *mOS* median overall survival, *ORR* objective response rate, *REF-ex* do not meet the REFLECT criteria, *REF-in* meet the REFLECT criteria

The outcomes of lenvatinib in REFLECT-in and REFLECT-ex patients were further analyzed based on liver function, tumor burden in the liver, and treatment regimens.

Patients with Child–Pugh grade A vs. those with Child–Pugh grades B or C showed a remarkably longer median OS (13.3 vs. 6.7 months; P = 0.0247) [44, 45, 47, 50, 51] and numerically better median PFS (6.8 vs. 4.9 months; P = 0.2854) [44, 45, 49–51] with similar ORR (42.5% vs. 42.9%; P = 0.9794) and DCR (80.3% vs. 79.7%; P = 0.9696) [49–52] (Table 3).

Patients with an extensive tumor burden in the liver (main portal vein invasion (MPVI), BDI, or tumor ≥ 50% liver occupation) achieved a median OS of 6.0 months and a median PFS of 3.9–4.0 months [50, 51]. Patients with MPVI vs. those without MPVI had numerically shorter median OS (10.2 vs. 13.8 months; P = 0.063) [47] and median PFS (5.6 vs. 9.9 months; P = 0.09) [49]. Similar trend for median OS was detected in patients with or without PVT (5.6 vs. 7.9 months) [44], with or without MVI (8.8 vs. 15.6 months; P = 0.003) [47], and with ≥ 50% or < 50% liver occupation (8.6 vs. 9.9 months; P = 0.49) [49]. Tumor responses were not significantly different in patients with or without MPVI (ORR: 54.3% vs. 40.0%; P = 0.5335; DCR: 91.5% vs. 100%; P = 0.3522), with or without BDI (ORR: 85.7% vs. 51.1%; P = 0.061; DCR: 100% vs. 91.3%; P = 0.2683), and with ≥ 50% or < 50% liver occupation (ORR: 33.3% vs. 56.3%; P = 0.1329; DCR: 91.7% vs. 92.0%; P = 0.9728) [49].

Lenvatinib was used as a second- or later-line therapy in real-world practice, and no significant difference was observed in patients with first-line or later-line lenvatinib in terms of median OS (13.3 months vs. not reached; P = 0.233), median PFS (5.1–9.1 vs. 5.1–11.9 months), ORR (44%–56.9% vs. 35%–44.4%), and DCR (68%–91.7% vs. 68%–92.6%) [49, 51]. In Child–Pugh A patients, first-line lenvatinib showed numerically better median OS (10.7 vs. 6.4 months; P = 0.728), median PFS (4.6 vs. 4.1 months; P = 0.427), significantly higher ORR (21.1 vs. 0%; P = 0.039), and simlar DCR (82.5% vs. 88.2%) compared with second- or later-line lenvatinib [50].

Overall, lenvatinib had comparable outcomes in REFLECT-in and REFLECT-ex patients. Child–Pugh grades and MVI remarkably influenced median OS but not tumor responses, whereas tumor burden and treatment history were not significantly associated with median OS, median PFS, and tumor responses.

### Donafenib

Donafenib, developed by Suzhou Zelgen Biopharmaceuticals, China, is a derivative of sorafenib with a trideuterated *N*-methyl group (Fig. 1) to improve the molecular stability [53]. Donafenib is a multi-kinase inhibitor of RAF-1, VEGFRs, PDGFR-β, FGFR-1, FLT-3, KIT, and RET [54] Fig. 2).

#### Key clinical trial of donafenib

In the randomized, controlled, phase 2/3 ZGDH3 study (NCT02645981) conducted between March, 2016 and April, 2018 at 37 centers across China, 668 aHCC patients naïve to systemic therapy were randomized (1:1) to receive 200 mg donafenib or 400 mg sorafenib twice daily. Donafenib vs. sorafenib had significantly improved median OS (12.1 vs. 10.3 months; HR: 0.83; 95% CI 0.70–0.99; P = 0.0245) but similar median PFS (3.7 vs. 3.6 months; HR: 0.91; 95% CI 0.76–1.09; P = 0.0570), median TTP (3.7 vs. 3.7 months; P = 0.1029), ORRs (4.6% vs. 2.7%; P = 0.2448), and DCRs (30.8% vs. 28.7%; P = 0.5532) (Table 1). The incidence of drug-related grade ≥ 3 AEs was significantly lower for donafenib than for sorafenib (38% vs. 50%; OR: 0.65; 95% CI 0.47–0.89; P = 0.0018) [42, 55]. The National Medical Products Administration (NMPA) of China approved donafenib in 2021 as a first-line therapy for aHCC based on the results of ZGDH3 study.

ZGDH3 study was conducted in China only, whereas the pivotal trials of lenvatinib and sorafenib were conducted globally. The median age of the patients and the percentages of HBV-positive patients were 53 and 90% in ZGDH3 study [55], 62 and 50% in REFLECT study [41], 66 and 18% in SHARP study [19], and 51 and 73% in Asia–Pacific study [20], respectively. Hence, donafenib could be a treatment option for HBV-positive Chinese patients, but biological and etiopathogenic considerations should be taken for patients from non-Asian regions with the ages > 60 [56].

## Second-line therapy

### Regorafenib

Regorafenib, a fluoro-derivative of sorafenib (Fig. 1) developed by Bayer, blocks the activity of multiple kinases including RET, KIT, B-Raf, Raf-1, B-Raf^V600E^, VEGFRs, FGFR-1, PDGFR-β, and TIE2 [57] (Fig. 2).

#### Key clinical trial of regorafenib

In the randomized, double-blinded, placebo-controlled, multicenter, phase 3 RESORCE study (NCT01774344) conducted from May, 2013 to Dec, 2015 at 147 sites in 19 countries across Asia, Australia, Europe, North America, and South America regions, aHCC patients (n = 573) progressed on sorafenib were randomized (2:1) to receive 160 mg regorafenib or placebo once daily for the first 3 weeks of each 4-week cycle. Compared with placebo, regorafenib remarkably improved median OS (10.6 vs. 7.8 months; HR: 0.63; 95% CI 0.50–0.79; P < 0.0001), median PFS (3.1 vs. 1.5 months; HR: 0.46; 95% CI 0.37–0.56; P < 0.0001), and TTP (3.2 vs. 1.5 months; HR: 0.44; 95% CI 0.36–0.55; P < 0.0001) [58]. The median OS from sorafenib initiation was 26.0 months for regorafenib and 19.2 months for placebo. Regorafenib provided not only an OS benefit over placebo irrespective of the last sorafenib dose (800 or < 800 mg) but also a TTP benefit over placebo irrespective of TTP on prior sorafenib [59]. CR, PR, SD, and PD were 1%, 10%, 54%, and 23% for regorafenib, and 0%, 4%, 32%, and 56% for placebo, respectively. Accordingly, regorafenib had a significantly better ORR (11% vs. 4%; P = 0.0047) and DCR (65% vs. 36%; P < 0.0001) than placebo (Table 1). The percentage of patients who experienced serious AEs were comparable for regorafenib and placebo (100% vs. 93%) [58]. The FDA approved regorafenib in 2017 as a second-line therapy for aHCC based on the results of RESORCE study.

#### The outcomes of regorafenib under real-world conditions

In real-world practices, regorafenib showed a pooled median OS, median PFS, TTP, ORR, and DCR of 14.3 months (95% CI 11.8–16.8) [60–66], 5.4 months (95% CI 2.6–8.1) [61–65, 67, 68], 4.8 months [62, 63, 66], 11.7% (95% CI 9.9–13.6) [61, 62, 65–68], and 58.5% (95% CI 40.8–76.2) [61, 62, 65–68], respectively. The median OS from sorafenib initiation was 28.7 months (95% CI 21.8–35.7) [60–62, 65–67] (Table 4). Patients with HCC recurrence after liver transplantation who received second-line regorafenib had a median OS of 12.9 months from regorafenib initiation and 38.4 months from sorafenib initiation [60]. Thereby, regorafenib was effective for aHCC in real-world setting.

**Table 4:** The outcomes of regorafenib under real-world conditions

Reference	Patient No	mOS (months)	Sequential mOS	PFS (months)	TTP (months)	ORR (%)	DCR (%)
Hou et al. [67]	41	NR	35.3	6.6	–	9.8	80.5
Iavarone et al. [60]	28	12.9	38.4	–	–	–	–
Lee et al. [61]	83	14.7	21.2	2.9	–	10.8	44.2
Lee et al. [62]	112	10.0	25.8	2.7	2.6	12.5	34.8
Ogasawara et al. [63]	44	17.3	–	6.9	6.9	–	–
Ren et al. [64]	43	17.0	–	11.0	–	–	–
Wang et al. [65]	38	12.4	25.3	3.7	–	13.2	71.1
Yoo et al. [68]	40	NR	NR	3.7	–	10.0	58.2
Zhu et al. [66]	93	15.9	26.3	–	5.0	14.0	62.4
Means (95% CI)		14.3 (11.8–16.8)	28.7 (21.8–35.7)	5.4 (2.6–8.1)	4.8 (-0.5–10.1)	11.7 (9.9–13.6)	58.5 (40.8–76.2)
Means ± SD		14.3 ± 2.7	28.7 ± 6.6	5.4 ± 3.0	4.8 ± 1.2	11.7 ± 1.8	58.5 ± 16.9

*DCR* disease control rate, *mPFS* median progression-free survival, *mOS* median overall survival, *mTTP* median time to progression, *NR* not reached, *ORR* objective response rate

The association between the dose and TTP of first-line sorafenib and the efficacy of second-line regorafenib was investigated. Sorafenib dose (800 or < 800 mg) had no significant influence on median OS (P = 0.172) and median PFS (8.0 vs. 5.7 months; P = 0.084) of regorafenib. The starting dose of regorafenib (160 or < 160 mg) had remarkable influence on median PFS (10.7 vs. 5.7 months; P = 0.006), but not on median OS (P = 0.615) [67]. Yoo et al. reported that TTP of sorafenib (≥ or < median) was significantly associated with median OS (14.7 or 4.7–9.9 months; P < 0.05) [62, 68] and median PFS (9.2 vs. 2.5 months; P = 0.003) of regorafenib [68], but Hou et al. showed that TTP of sorafenib (≥ or < median) was not relevant to median OS of regorafenib (P = 0.552) [67]. TTPs of sorafenib (> or ≤ median) and regorafenib were not significantly correlated (7.7 vs. 4.1 months; P = 0.38) in Ogasawara’s study [63], but were positively associated (P < 0.001) in Lee’s study [62]. Major regorafenib-associated AEs observed in these studies were similar to those related to sorafenib.

Overall, the outcomes of regorafenib in real-world setting was comparable with those in RESORCE study. The controversial results regarding the relationship between sorafenib TTP and the survival outcomes of regorafenib were probably due to the differences in patient characteristics such as age, sex, HBV infection, and BCLC stages.

### Cabozantinib

Cabozantinib (Fig. 1), developed by Exelixis Inc. USA, is a multi-kinase inhibitor of MET, VEGFRs, AXL, RET, ROS1, TYRO3, MER, KIT, TRKB, FLT-3, and TIE-2 [69, 70] (Fig. 2).

#### Key clinical trial of cabozantinib

In the randomized, double-blind, controlled, phase 3 CELESTIAL study (NCT01908426) conducted between September, 2013 and October, 2017 at 104 sites across Asia–Pacific, Europe, North Amerinca, and Oceania regions, aHCC patients (n = 707) with prior sorafenib treatment and Child–Pugh A liver cirrhosis were randomized (2:1) to received 60 mg cabozantinib or placebo once daily. Compared with placebo, cabozantinib had notably improved median OS (10.2 vs. 8.0 months; HR: 0.0.76; 95% CI 0.63–0.92; P = 0.005), median PFS (5.2 vs. 1.9 months; HR: 0.44; 95% CI 0.36–0.52; P < 0.001), TTP (5.4 vs. 1.9 months; HR: 0.41; 95% CI 0.34–0.49), ORR (4% vs. 0.4%; P = 0.009), and DCR (64% vs. 33%; P < 0.001) (Table 1). AEs of any grade were 99% for cabozantinib and 92% for placebo, and the most common grade 3 or 4 AEs for cabozantinib were PPE, hypertension, increased aspartate aminotransferase level, fatigue, and diarrhea [71, 72]. The FDA approved cabozantinib in 2019 as a second-line therapy for aHCC based on the results of CELESTIAL study.

Subgroup analyses of CELESTIAL data were performed based on liver function, disease etiology, disease extension, prior treatments, and baseline alpha-fetoprotein (AFP) levels.

In Child–Pugh A patients, cabozantinib prolonged median OS and median PFS vs. placebo in both ALBI grade 1 (17.5 vs. 11.4 months; HR: 0.63; 95% CI 0.46–0.86 and 6.5 vs. 1.9 months; HR: 0.42, 95% CI 0.32–0.56) and grade 2 (8.0 vs. 6.4 months; HR: 0.84; 95% CI 0.66–1.06 and 3.7 vs. 1.9 months; HR: 0.47; 95% CI 0.37–0.58) subgroups. ORR and DCR were greater with cabozantinib vs. placebo in both ALBI grade 1 (4% vs. 1% and 74% vs. 40%) and grade 2 (4% vs. 0% and 57% vs. 28%) subgroups [73]. In patients whose liver cirrhosis deteriorated to Child–Pugh B by week 8, cabozantinib vs. placebo improved median OS (8.5 vs. 3.8 months; HR: 0.32; 95% CI 0.18–0.58) and median PFS (3.7 vs. 1.9 months; HR: 0.44; 95% CI 0.25–0.76). Neither cabozantinib nor placebo achieved ORR, but cabozantinib had higher SD (57% vs. 23%) and lower PD (41% vs. 68%) than placebo [74].

In HBV-positive patients (with or without HCV infection), cabozantinib extended median OS (9.7 vs. 6.1 months; HR: 0.69, 95% CI 0.51–0.94) and median PFS (4.4 vs. 1.8 months; HR: 0.31, 95% CI 0.23–0.42) compared with placebo. In HCV-positive patients (without HBV infection), cabozantinib vs. placebo had comparable median OS (11.1 vs. 11.4 months; HR: 1.11, 95% CI 0.72–1.71) but better median PFS (4.1 vs. 1.9 months; HR: 0.61, 95% CI 0.42–0.88) [71, 75].

In patients with EHS, cabozantinib had better median OS (9.6 vs. 6.9 months; HR: 0.72; 95% CI 0.58–0.89) and median PFS (5.0 vs. 1.9 months; HR: 0.46; 95% CI 0.37–0.56) than placebo. When MVI was present, patients on cabozantinib and placebo achieved a median OS of 7.6 and 5.3 months (HR: 0.75; 95% CI 0.54–1.03) and a median PFS of 3.7 and 1.8 months (HR: 0.42; 95% CI 0.31–0.58) respectively. In patients with EHS and/or MVI, cabozantinib extended median OS (9.5 vs. 7.3 months; HR: 0.73; 95% CI 0.60–0.90) and median PFS (5.0 vs. 1.9 months; HR: 0.45; 95% CI 0.37–0.54) compared with placecbo. In patients with neither EHS nor MVI, cabozantinib vs. placebo had comparable median OS (14.0 vs. 14.7 months; HR: 0.99; 95% CI 0.59–1.65) but longer median PFS (5.6 vs. 2.0 months; HR: 0.46; 95% CI 0.29–0.74) [71, 75].

In patients received sorafenib as the only prior systemic therapy, cabozantinib extended median OS (11.3 vs. 7.2 months; HR: 0.70; 95% CI 0.55–0.88) and median PFS (5.5 vs. 1.9 months; HR: 0.40; 95% CI 0.32–0.50) compared with placebo. Both ORR (all responses were PR) and SD were higher with cabozantinib than with placebo (5% vs. 0.6% and 62% vs. 30%). Such improved outcomes of cabozantinib over placebo were irrespective of duration of prior sorafenib treatment (< 3, 3 to < 6, and ≥ 6 months), and longer durations of prior sorafenib generally corresponded to longer median OS and median PFS [76].

High serum levels of AFP are associated with poor prognosis in HCC patients. CELESTIAL data were analyzed by baseline AFP levels with a cut-off of 400 ng/mL and by AFP response (≥ 20% decrease from baseline at week 8). Cabozantinib improved median OS and median PFS vs. placebo in patients with baseline AFP < 400 ng/mL (13.9 vs. 10.3 months; HR: 0.81; 95% CI 0.62–1.04 and 5.5 vs. 1.9 months; HR: 0.47, 95% CI 0.37–0.60) as well as in patients with baseline AFP ≥ 400 ng/mL (8.5 vs. 5.2 months; HR: 0.71; 95% CI 0.54–0.94 and 3.9 vs. 1.9 months; HR: 0.42; 95% CI 0.32–0.55). ORR was higher with cabozantinib than with placebo in patients with baseline AFP < 400 ng/mL (5% vs. 0.7%) and ≥ 400 ng/mL (3% vs. 0%). AFP response rate was 50% for cabozantinib and 13% for placebo. In the cabozantinib arm, AFP responders had better median OS (16.1 vs. 9.1 months; HR: 0.61; 95% CI 0.45–0.84), median PFS (7.3 vs. 4.0 months; HR: 0.55; 95% CI 0.41–0.74), higher ORR (7% vs. 3%), and lower SD (15% vs. 29%) than non-responders [77].

Altogether, cabozantinib was beneficial for aHCC patients regardless of liver function, HBV or HCV infection, disease extension, the duration of prior sorafenib treatment, and baseline AFP levels, further supporting the utility of cabozantinib as a second-line therapy for aHCC.

#### Outcomes of cabozantinib in retrospective and real-life studies

Cabozantinib as a second- or later-line therapy for HCC had a pooled median OS, median PFS, TTP, ORR, and DCR of 7.8 months (95% CI 4.3–11.3) [78–82], 3.7 months (95% CI 2.7–4.8) [78–81, 83], 3.88–5.2 months [81, 82], 5.1% (95% CI 3.2–7.0) [78–83], and 55.9% (95% CI 43.7–68.1) [78–83], respectively (Table 5). Second-line cabozantinib had comparable median OS vs. third- or later-line cabozantinib (8.1 vs. 7.0 months; P = 0.88) [79]. Third- or earlier-line cabozantinib showed similar median OS (3.2 vs. 4.3 months; P = 0.708) but numerically better PFS compared with fourth- or later-line cabozantinib (9.2 vs. 3.7 months; P = 0.148) [80].

**Table 5:** The outcomes of cabozantinib in retrospective and real-life studies

Reference	Patient No	mOS (months)			mPFS (months)			TTP (months)		ORR (%)	DCR (%)	
		Overall	CPA vs. CPB	AFP ≥ 400 vs. < 400 ng/mL	Overall	CPA vs. CPB	AFP ≥ 400 vs. < 400 ng/mL	Overall	CPA vs.CPB		Overall	CPA vs. CPB
Bang et al. [78]	110	7.5	9.0 vs. 3.8 (P < 0.001)	5.3 vs. 9.5 (P = 0.014)	3.7	4.3 vs. 2.2 (P < 0.001)	3.1 vs. 4.6 (P = 0.14)	–	–	3.6	66.3	71.5 vs. 45.5 (P = 0.021)
Finkelmeier et al. [79]	88	6.8	9.7 vs. 3.4	4.9 vs. 9.7 (P = 0.054)	3.2	–	–	–	–	7.0	39.0	–
Kanzaki et al. [80]	23	4.3	6.9 vs. 2.1 (P < 0.001)	–	3.7	9.2 vs. 0.5 (P = 0.001)	–	–	2.1 vs. 0.5 (P < 0.001)	4.4	56.6	–
Tomonari et al. [83]	26	–	–	–	3.0	–	–	–	–	7.7	65.4	–
Tovoli et al. [81]	96	12.1	–	–	5.1	–	–	5.2	–	4.2	63.5	–
Wong et al. [82]	27	8.28	–	–	–	–	–	3.88	–	3.7	44.4	–
Means (95% CI)		7.8 (4.3–11.3)	8.5 (4.9–12) vs. 3.1 (0.89–5.3) (P = 0.0053)	–	3.7 (2.7–4.8)	–	–	–	–	5.1 (3.2–7.0)	55.9 (43.7–68.1)	–
Means ± SD		7.8 ± 2.8	(8.5 ± 1.5) vs. (3.1 ± 0.9)	–	3.7 ± 0.8	–	–	–	–	5.1 ± 1.8	55.9 ± 11.6	–

*AFP* alpha-fetoprotein, *CPA* Child–Pugh grade A, *CPB* Child–Pugh grade B, *DCR* disease control rate, *mPFS* median progression-free survival, *mOS* median overall survival, *ORR* objective response rate, *TTP* time to progression

The outcome of cabozantinib was further analyzed in stratified HCC patients.

Cabozantinib at full-dose (60 mg daily) and reduced-dose (20 or 40 mg daily) had comparable median PFS (2.8 vs. 3.6 months; P = 0.59), ORR (6.7% vs. 9.1%; P = 1) and DCR (53.4% vs. 81.8%; P = 0.22). However, the incidence of AEs, such as decreased appetite, fatigue, and diarrhea, was significantly higher in the full-dose group than in the reduced-dose group for all grades (P < 0.05) [83].

Cabozantinib showed a favorible tendency for patients at BCLC stage A or B vs. those at BCLC stage C in terms of median OS (14.1 vs. 6.6 months; P = 0.056) [79]. Child–Pugh A patients had significantly better median OS (8.5 vs. 3.1 months; P = 0.0053) [78–80], median PFS (4.3–9.2 months vs. 0.5–2.2 months; P ≤ 0.001) [78, 80], TTP (2.1 vs. 0.5 months; P < 0.001) [80], and DCR (71.5% vs. 45.5%; P = 0.021) [78] compared with Child–Pugh B patients (Table 5). Patients with ALBI grade 1 or 2 showed significantly longer OS (13 vs. 5.6 months; P < 0.001) and PFS (6.2 vs. 3.5 months; P = 0.009) than those with ALBI grade 3 [78].

Patients with AFP levels ≥ 400 ng/mL had a trend toward worse OS (4.9–5.3 months vs. 9.5–9.7 months) [78, 79] and PFS (3.1 vs. 4.6 months; P = 0.14) [78] vs. those with AFP levels < 400 ng/mL (Table 5). The most common AEs were HFSR, thrombocytopenia, diarrhea, fatigue, and anorexia [78, 79].

Overall, the efficacy of cabozantinib as second- or later-line therapy in real‐world setting was comparable to those in CELESTIAL study. The outcomes of cabozantinib were significantly associated with liver function but not BCLC stage and baseline AFP levels. In addition, dose reduction may be a safer treatment option for aHCC.

### Apatinib

Apatinib, a derivative of valantinib (Fig. 1) developed by Jiangsu Hengrui Medicine, China, blocks the activities of VEGFR-2, KIT, RET, and c-Src (Fig. 2). The affinity of apatinib for VEGFR-2 is ten times of that of sorafenib [84, 85].

#### Key clinical trial of apatinib

In the randomized, double blind, placebo-controlled, multicenter phase 3 AHELP study (NCT02329860) conducted between April, 2014 and May, 2017 in China, aHCC patients (n = 400) who have progressed on systemic therapy were randomly assigned (2:1) to receive 750 mg apatinib or placebo once daily in 4-week cycles. Apatinib remarkably extended the median OS (8.7 vs. 6.8 months; P = 0.048), median PFS (4.5 vs. 1.9 months; P < 0.0001), and median TTP (4.7 vs. 1.9 months; P < 0.0001) compared with placebo. CR was not achieved in either group, PR, SD, and PD were 11%, 51%, and 18% for apatinib, and 2%, 27%, and 55% for placebo, respectively. Accordingly, apatinib had higher ORR (11% vs. 2%) and DCR (61% vs. 29%) than placebo (Table 1). The most common treatment-related AEs of grade 3 or 4 were hypertension, hand-foot syndrome (HFS), and decreased platelet count in the apatinib group [86]. The NMPA of China approved apatinib in 2020 as a second-line therapy for aHCC based on the results of AHELP study [87].

#### The outcomes of apatinib under real-world conditions

In patients received apatinib as a first-line, second-line, or third-line therapy, median OS was 16.0, 17.0, and 5.8 months, and median PFS was 8.2, 7.0, and 3.1 months, respectively. Patients with first- and second-line apatinib had significantly prolonged median OS (P < 0.001) and median PFS (P < 0.001) than those with third-line apatinib. The most common AEs were secondary hypertension, gastrointestinal resistance, fatigue, and HFS, which were tolerable and manageable [88].

In patients with unresectable or relapsed HCC, apatinib had a median OS of 13 months and a median PFS of 5 months, and the most common AEs were proteinuria, secondary hypertension, and liver dysfunction [89].

In patients with sorafenib-refractory aHCC, apatinib (500 mg once daily) had a median OS of 8 months and a median TTP of 3 months. No patients achieved CR, ORR was 25.6%, and DCR was 67.4%. Patients with PR (25.6%), SD (41.9%), and PD (32.6%) had a median OS of 19, 8, and 4 months, and a median TTP of 14, 3, and 1 month(s), respectively [90].

Apatinib significantly improved median PFS (6.3 vs. 2.5 months; P < 0.001) and ORR (21.4% vs. 5%; P = 0.019) in HBV-positive aHCC patients with lung metastasis only vs. those with non-lung metastasis, and median OS tended to be better in the lung metastasis group than in the non-lung metastasis group (17.5 vs. 8.3 months; P = 0.346) [91]. In HBV-positive patients with sorafenib resistant aHCC, apatinib was associated with longer median OS (7.0 vs. 4.0 months; P < 0.001) compared with supportive care. ORR and DCR were 22.4% and 55.2% respectively [92].

These studies provide evidence that apatinib is not only effective as both first-line and second-line therapy for HCC patients, but also promising in treating patients (infected or not with HBV) with sorafenib refractory or resistant aHCC.

## Comparison of the outcomes of approved small molecule TKIs for aHCC

As aforementioned, patients with aHCC can benefit from first-line sorafenib and lenvatinib, and those who progressed on sorafenib can benefit from second-line regorafenib and cabozantinib. In addition, apatinib was used as a first-line treatment for aHCC in both clinial trial and real-world setting although it was approved as a second-line therapy. To find out which TKI is more effective for aHCC, the clinical outcomes of lenvatinib vs. sorafenib, apatinib vs. sorafenib, and regorafenib vs. cabozantinib were compared.

### Lenvatinib vs. sorafenib

Meta-analyses were conducted to compare the efficacy and safety of lenvatinib vs. sorafenib as first-line therapy for aHCC.

Facciorusso et al. analyzed 5 studies containing 1481 patients and found that lenvatinib vs. sorafenib had comparable median OS (13.4 vs. 11.4 months; HR: 0.81; 95% CI 0.58–1.11; P = 0.19) but significantly better median PFS (5.88 vs. 4.17 months; HR: 0.67; 95% CI 0.48–0.94; P = 0.02), ORR (33.3% vs. 6.5%; OR: 7.7; 95% CI 2.99–19.82; P < 0.0001), and DCR (76.9% vs. 52.7%; OR: 2.41; 95% CI 1.55–3.77) [93]. Similar results were reported by Luo et al. who systematically reviewed 15 studies comprising 3908 patients and showed that lenvatinib vs. sorafenib had comparable OS (HR: 0.86; 95% CI 0.72–1.02; P = 0.09) but remarkably better PFS (HR: 0.63; 95% CI 0.53–0.74; P < 0.00001), ORR (OR: 5.61; 95% CI 3.90–8.09; P < 0.00001), and DCR (OR: 2.42; 95% CI 1.79–3.28; P < 0.00001) [94].

Wang et al. analyzed 12 studies containing 3510 patients and revealed that lenvatinib was associated with numerically better OS (HR: 0.85; 95% CI 0.73–0.99; P = 0.075), PFS (HR: 0.72; 95% CI 0.61–0.84; P = 0.066), DCR (OR: 2.23; 95% CI 1.70–2.93; P = 0.098), and significantly higher ORR (OR: 4.25; 95% CI 2.78–6.48; P = 0.045) compared with sorafenib [95]. Similarly, Hu et al. conducted a meta-analysis of 9 studies comprising 1914 patients and found that lenvatinib vs. sorafenib had improved OS (HR: 0.87; 95% CI 0.78–0.97), PFS (HR: 0.67; 95% CI 0.48–0.94), ORR (OR: 7.44; 95% CI 4.44–12.46), and DCR (OR: 4.57; 95% CI 2.3–9.07) [96].

The severe AE rate was similar in the lenvatinib and sorafenib groups [93–95], but lenvatinib was significantly associated with a higher incidence of hypertension, proteinuria, fatigue, decreased appetite, and weight loss, whereas sorafenib was associated with a higher incidence of diarrhea and HFSR [94, 96].

These meta-analyses demonstrated that lenvatinib provided better tumor responses and survival advantages over sorafenib as a first-line treatment for aHCC, with a comparable incidence of severe AEs.

### Apatinib vs. sorafenib

In an open-label, single-arm phase 2 trial (NCT03046979) conducted between December, 2016 and June, 2018 in China, aHCC patients (n = 23) who received first-line apatinib (500 mg once daily) had a median OS of 13.8 months (95% CI 5.3–22.3) and a median PFS of 8.7 months (95% CI 5.9–11.1). ORR and DCR were 30.4% and 65.2% respectively. The most common treatment-related AEs were proteinuria, hypertension, and HFSR [97]. Apatinib was therefore beneficial for aHCC as a first-line treatment.

The effectiveness of first-line apatinib for aHCC was compared with sorafenib in retrospective studies.

aHCC patients on apatinib vs. those on sorafenib showed comparable median PFS (4.1 vs. 3.6 months; P = 0.925), median OS (HR: 1.15; CI 0.369–3.58; P = 0.811), DCR (57.7% vs. 50%; P = 0.530), and siginifcanlty higher ORR (19.2% vs. 2.2%; P = 0.012). The most common any-grade AEs in the entire patient cohort were elevated transaminase, HFS, and diarrhea, but more patients in the apatinib group experienced with hypertension compared with those in the sorafenib group (50% vs. 19.6%) [98].

Patients with intermediate or advanced HCC who received sorafenib vs. those received apatinib achieved significantly longer median OS (10.4 vs. 7.18 months; P = 0.011) and median PFS (7.39 vs. 4.79 months; P = 0.031), whereas ORR (15.78% vs. 17.64%; P = 0.833) and DCR (42.1. % vs. 38.2%; P = 0.75) were similar for sorafenib and apatinib. HFS and diarrhea were the common AEs in the sorafenib group, whereas hypertension, proteinuria, and increased transaminase were the common AEs in the apatinib group [99].

Further analysis of the above two studies revealed that apatinib was not superior to sorafenib in terms of ORR (risk ratio (RR): 1.99; 95% CI 0.85–4.65; P = 0.111) and DCR (RR: 1.04; 95% CI 0.73–1.47; P = 0.840) but was inferior to sorafenib in the survival rates of 6-month (RR: 0.63; 95% CI 0.42–0.97; P = 0.036) and 1-year (RR: 0.47; 95% CI 0.29–0.79; P < 0.0001) [100]. Sorafenib appeared to be a better option for aHCC as a first-line therapy compared with apatinib.

### Regorafenib vs. cabozantinib

The clinical outcomes of cabozantinib and regorafenib as second-line therapy for aHCC patients with prior sorafenib were compared by matching-adjusted indirect comparison.

Patients received cabozantinib in CELESTIAL study and those received regorafenib in RESORCE study had similar median OS (11.4 vs. 10.6 months; P = 0.3474), whereas median PFS was notably longer for cabozantinib than for regorafenib (5.6 vs. 3.1 months; P = 0.0005) [101].

The median OS for cabozantinib in CELESTIAL study and for regorafenib in a real-world practice was comparable (11.3 vs. 11.1 months; HR: 0.83, 95% CI 0.62–1.09), but cabozantinib had better median OS compared with regorafenib in patients with prior sorafenib treatment < 6 months. Median PFS was longer for cabozantinib than for regorafenib (5.5 vs. 3.0 months; HR: 0.50; 95% CI 0.41–0.62), and the prolonged median PFS of cabozantinib over regorafenib was irrespective of the duration of prior sorafenib treatment (< 3, 3 to < 6, and ≥ 6 months) [102]. However, Adhoute et al. reported that cabozantinib and regorafenib had similar median PFS (3.6 vs. 2.9 months; P = 0.7986) based on retrospective data obtained from three French centers [103]. The inconsistent PFS data were probably due to different cohort size in the two studies. No significant difference was found in the regorafenib and cabozantinib arms in terms of frequency of grade 3 or 4 treatment emergent AEs [101, 103].

These studies revealed that regorafenib and cabozantinib had no significant differences in terms of OS, and earlier progressors on prior sorafenib may benefit more from cabozantinib treatment.

Altogether, lenvatinib was more effective than sorafenib as a first-line therapy for aHCC in terms of survival and tumor responses, whereas apatinib was not as effective as sorafenib as a first-line treatment option although it significantly improved the clinical outcomes of aHCC patients. The second-line regorafenib and cabozantinib had comparable OS, but aHCC patients with early progress on sorafenib may benefit more from cabozantinib.

## Future perspectives

Currently, the six small molecule TKIs are the main therapeutic options for aHCC, however, the prognosis of aHCC patients is not optimistic due to the development of resistance to these TKIs [104]. In addition, severe AEs caused by these TKIs require discontinuation of the treatment. To circumvent drug resistance and improve the therapeutic outcomes of TKIs, large efforts have been made to develop novel TKIs and TKI-based combination therapies.

### Development of novel TKIs

In an decade following the approval of sorafenib for aHCC, a number of small molecule TKIs were developed. Sunitinib [105], linifanib (ABT-869) [106], erlotinib [107], brivanib [107], and tivantinib (ARQ-197) [108] were evaluated in phase 3 trials for systemic therapy of aHCC. Unfortunately, these TKIs failed to show meaningful improvement in aHCC patients compared with sorafenib or placebo [109–115]. Axitinib vs. placebo improved PFS and TTP but did not prolong OS in patients with advanced, metastatic, or inoperable HCC [116]. Anlotinib showed promising efficacy and safety as a first- or second-line treatment for aHCC in clinical trials and real-world study [117–119]. A phase 3 trial (NCT03950518) and an observational study (NCT04954521) are ongoing to evaluate the clinical outcomes of anlotinib in aHCC patients (Table 6).

**Table 6:** Ongoing clinical trials of TKIs and TKI-based combination therapies for advanced hepatocellular carcinoma

Study (trial identifer)	Estimated enrollment	Trial phase	Study arm	Setting	Primary outcome	Estimated study duration
NCT03950518	300	3	Anlotinib vs. anlotinib + arginine vs. anlotinib + arginine + levamisole	Second-line	PFS	04/2019 04/2023
NCT04954521	200	Real-world	Anlotinib	–	OS	06/2021 07/2024
SOURCE (NCT04143191)	158	3	Sorafenib + TACE vs. sorafenib	First-line	RFS	09/2019 09/2023
SEARCH (NCT05718232)	136	3	Lenvatinib + SBRT + TACE vs. lenvatinib	First-line	OS	03/2023 02/2027
SOLARIS (NCT05101629)	32	2	Pembrolizumab (PD-1 mAb) + lenvatinib	Second-line	ORR	05/2022 12/2024
NCT04728321	32	2	AK104 (PD-1/CTLA-4 bispecitic mAb) + lenvatinib vs. AK104	First-line	ORR	01/2021 03/2023
NCT04194775	534	3	Nofazinlimab (CS1003; PD-1 mAb) + lenvatinib vs. placebo + lenvatinib	First-line	OS	12/2019 06/2025
NCT05168163	122	2	Atezolizumab (PD-L1 mAb) + lenvatinib vs. lenvatinib Atezolizumab + cabozantinib vs. cabozantinib	Second-line	OS, PFS	05/2022 12/2026
NCT04770896	554	3	Atezolizumab (PD-L1 mAb) + lenvatinib vs. lenvatinib Atezolizumab + sorafenib vs. sorafenib	Second-line	OS	04/2021 09/2024
NCT03211416	37	1/2	Sorafenib + pembrolizumab (PD-1 mAb)	First- or second line	ORR	12/2017 10/2023
NCT03439891	16	2	Sorafenib + nivolumab (PD-1 mAb)	First-line	MTD, ORR	04/2018 11/2024
NCT03764293	543	3	Camrelizumab (PD-1 mAb) + apatinib vs. sorafenib	First-line	OS, PFS	06/2019 07/2023
NCT04696055	95	2	Regorafenib + pembrolizumab (PD-1 mAb)	Second-line	ORR	02/2021 05/2024
NCT05048017	20	2	Regorafenib + PD-1 mAb (camrelizumab, toripalimab, and pembrolizumab)	Second-line	PFS	10/2021 12/2025
GOING (NCT04170556)	78	1/2	Regorafenib + nivolumab (PD-1 mAb)	Second-line	AEs	03/2020 08/2024
NCT04183088	125	2	Part 1: regorafenib + tislelizumab (PD-1 mAb) Part 2: regorafenib + tislelizumab vs. regorafenib	First-line	AEs, ORR, PFS	12/2020 03/2026
NCT04344158	648	3	Anlotinib + AK105 (penpulimab; PD-1 mAb) vs. sorafenib	First-line	OS	05/2020 12/2024
FAITH (NCT06031480)	55	2	Anlotinib + TQB2450 (PD-L1 mAb)	Second-line	ORR	10/2023 12/2025

*AEs* adverse effects, *CTLA-4* T-lymphocyte-associated antigen 4, *MTD* maximum tolerated dose, *OS* overall survival, *ORR* objective response rate, *PD-1* programmed cell death protein 1, *PD-L1* programmed cell death ligand 1, *PFS* progress-free survival, *RFS* recurrence-free survival, *SBRT* stereotactic body radiotherapy, *TACE* transarterial chemoembolization, *TKI* tyrosine kinase inhibitor

With the advancement of computing and technology in recent times, the conventional methods of drug design, a time-consuming, expensive, and complex process, has been replaced by artificial intelligence (AI). Accumulated evidence has shown that AI can increase the success rate of the designed drug and reduce the cost substantially [120]. It is conceivable that AI will accelerate the development of novel TKIs with potential effecfiveness for the therapy of aHCC.

### TKI-based combination therapies

#### TKIs in combination with locoregional therapy modalities

In clinical practice, small molecule TKIs were added to various locoregional therapy (LRT) modalities including transarterial chemoembolization (TACE), hepatic arterial infusion chemotherapy (HAIC), and stereotactic body radiotherapy (SBRT) for the treatment of HCC. It has been documented that lenvatinib plus TACE [121], HAIC [122], or SBRT [123, 124], sorafenib plus HAIC [125] or SBRT [126], and apatinib [127], anlotinib [128], axitinib [129], or sunitinib [130] plus TACE improved the survival outcomes of HCC patients compared with lenvatinib [121, 123], sorafenib [125], TACE [122, 127, 128, 130], or SBRT [124, 126] alone. These results remain to be confirmed by large-scale randomized controlled trials (RCTs). At present, sorafenib in combination with TACE is not recommended for the treatment of HCC, but a phase 3 trial (NCT04143191) is ongoing to explore the efficacy and safety of sorafenib plus TACE vs. sorafenib monotherapy for resectable aHCC. Another phase 3 trial (NCT05718232) is ongoing to evaluate the effectiveness of SBRT combined with TACE and lenvatinib vs. lenvatinib alone in aHCC patients with PVTT (Table 6).

#### TKIs in combination with immune checkpoint inhibitors

Immune checkpoints, such as programmed cell death protein 1 (PD-1), programmed cell death ligand 1 (PD-L1), and cytotoxic T-lymphocyte-associated antigen 4 (CTLA-4), are membrane bound proteins that prevent the overreaction of immune response and induce immune tolerance. Immune checkpoint inhibitors (ICIs) have brough a revolution in cancer therapy within the past 10 years [131], and the clinical outcomes of ICIs vs. TKIs in aHCC were investigated by clinical trials and real-world studies.

In the phase 3 IMbrave150 study (NCT03434379), combination of monoclonal antibodies (mAbs) against atezolizumab (PD-L1) and bevacizumab (VEGF mAb) outperformed sorafenib in patients with locally advanced or metastatic HCC naïve to systemic treatment in terms of median OS, median PFS, and ORR, whereas grade 3 or 4 AEs were comparable in the two groups [132, 133]. The FDA approved atezolizumab combining bevacizumab as first-line therapy for aHCC in 2020 [134].

The clinical outcomes of atezolizumab plus bevacizumab vs. lenvatinib in patients with unresectable HCC (uHCC) were compared by meta-analyses. Atezolizumab plus bevacizumab resulted in better PFS [135], comparable OS [136, 137] and PFS [137], or worse OS and PFS [136] compared with lenvatinib. In non-viral aHCC patients, lenvatinib was associated with signinficantly improved OS and PFS compared to atezolizumab plus bevacizumab [138], but ORR and DCR were not significantly different in the two treatment groups [135–137]. Different types of studies (e.g. real-world, retrospective, or prospective studies) and different patient sub-populations included in these studies may result in the discrepant PFS and OS data.

In the phase 3 HIMALAYA study (NCT03298451), tremelimumab (CTLA-4 mAb) plus durvalumab (PD-L1 mAb) surpassed sorafenib in patients with uHCC and no previous systemic treatment in terms of median OS, whereas durvalumab alone had a median OS that was noninferior to sorafenib. Median PFS was not signigicantly different among the three groups [139]. The 4-year follow-up study revealed the outperformance of tremelimumab plus durvalumab over sorafenib in terms of long-term OS [140]. The FDA approved tremelimumab combining durvalumab as first-line therapy for uHCC in 2022 [141].

In aHCC patients with prior sorafenib treatment, pembrolizumab (PD-1 mAb) vs. placebo achieved numerically better median OS and median PFS in the phase 3 KEYNOTE-240 study (NCT02702401) [142], and nivolumab (PD-1 mAb) plus ipilimumab (CTLA-4 mAb) showed promising results in the pase 1/2 CheckMate 040 study (NCT01658878) [143]. The FDA approved pembrolizumab and nivolumab plus ipilimumab as second-line therapy for aHCC in 2018 and 2020 respectively [134, 141].

Although ICIs remarkably improved the survival of HCC patients, only 30%–40% patients were responsive to ICIs, and responders may eventually develop resistance to ICIs [144]. To improve the clinical benefits of ICIs and overcome drug resistance, various combinations of ICIs and small molecule TKIs have been explored in clincal trials and real-world studies.

Lenvatinib plus nivolumab vs. lenvatinib significantly improved median OS and ORR in aHCC patients in a real-world study [145], but lenvatinib plus pembrolizumab failed to show superiority over lenvatinib monotherapy [146]. Apatinib plus camrelizumab (PD-1 mAb) showed promising efficacy and manageable AEs in aHCC patients as both first-line and second-line therapy [147], anlotinib plus sintilimab (PD-L1 mAb) and axitinib plus avelumab (PD-L1 mAb) had promising clinical activities with manageable toxicity as first-line treatment of aHCC [148, 149], but cabozantinib plus atezolizumab vs. sorafenib did not provide significant OS benefit [150]. The results remain to be confirmed by large-scale RCTs. So far a slew of ongoing clinical trials are evaluating the efficacy and safety of small molecule TKIs in combination with ICIs as first- or second-line therapies for aHCC (Table 6). These clinical trials will provide valuable information for TKI-based immunotherapy for aHCC.

#### Identification of predictive biomarkers for the therapeutic effects of TKIs

Large efforts have been made to identify potential biomarkers to predict the outomes of TKIs in HCC. In patients treated with sorafenib, high expression of Ang-2, a pro-angiogenic cytokine, was associated with poor survival [151], whereas a low neutrophil-to-lymphocyte ratio (NLR) that reflects the inflammatory response to cancer [152], and high expression of miR-224 [153] and miR-425-3p [154] in tumor samples were associated with better survival. Lenvatinib was shown to have a higher selectivity for FGFR compared with other TKIs, and the results from HCC cell lines suggested that amplification of FGF19 and FGFR might be biomarkers for lenvatinib effectiveness [155]. In patients treated with lenvatinib, higher NLR was associated with poor prognosis [156]. However, these results need further validation. As a result, there are still no predictive biomarkers for the clinical outcomes of TKI in HCC.

With the fully utilization of the advanced technologies in clinical settings, such as the analysis of tumor genomic or transcriptomic profiles, miRNA evaluation, the identification of driver gene mutations, ctDNA detection, molecular imagining, and cell cultures, more options will be available to identify predictive biomarkers for TKI treatment, leading to further improvement of the therapeutic effects of TKIs [157].

## Conclusion

Small molecule TKIs improved the clinical outcomes of HCC patients in both clinical trials and real-world setting. The efficacy and safety of TKIs may associate with race, age, sex, body weight, drug dose, the duration of treatment, baseline AFP levels, disease etiology, BCLC stage, liver function, tumor burden in the liver, and disease extension, which should be considered when choosing a treatment option for aHCC.

The advancement in AI will facilitate the development of novel TKIs with potential effectiveness for aHCC. Clinical trials and real-life studies revealed that TKIs in combination with LRTs or ICI inhibitors might be potential treatment options for aHCC. A large number of clinical trials are ongoing to investigate the clinical outcomes of novel TKI-based combination therapies, with considerations of dosing schedule, treatment sequence, duration, and treatment history. Furthermore, identification of the predictive biomarkers for TKIs treatment will improve the therapeutic effects this class of agents.

Altogether, the development of novel small molecule TKIs and novel TKI-based combination therapies, along with the identification of the predictive biomarkers for TKIs treatment, are promising in providing more treatment options and survival benefits for aHCC in the future.

## Data Availability

No datasets were generated or analysed during the current study.

## Abbreviations

AEs: Adverse events
aHCC: Advanced hepatocellular carcinoma
ALBI: Albumin-bilirubin
BCLC: Barcelona Clinic Liver Cancer
BDI: Bile duct invasion
CSF-1R: Colony-stimulating factor 1 receptor
c-Met: Hepatocyte growth factor receptor
CR: Complete response
DCR: Disease control rate
EGFR: Epithelial growth factor receptor
EHS: Extrahepatic spread
FDA: Food and Drug Administration
FGFR: Fibroblast growth factor receptor
FLT-3: Tumorigenic Fms-like tyrosine kinase 3
HBV: Hepatitis B virus
HCV: Hepatitis C virus
HFS: Hand and foot syndrome
HFRR: Hand-foot skin reaction
HAIC: Hepatic arterial infusion chemotherapy
HR: Hazard ratio
KIT: Stem cell factor receptor
mRECIST: Modified Response Evaluation Criteria in Solid Tumors
MPVI: Main portal vein invasion
MVI: Macrovascular invasion
NLR: Neutrophil-to-lymphocyte ratio
NMPA: National Medical Products Administration
OR: Odds ratio
ORR: Objective response rate
OS: Overall survival
PD: Progerssive disease
PDGFR: Platelet-derived growth factor receptor
PFS: Progression-free survival
PR: Partial response
PVT: Portal vein thrombosis
RCTs: Randomized controlled trials
RR: Risk ratio
RECIST: Response Evaluation Criteria in Solid Tumors
RET: Rearranged during transfection
RTKs: Receptor tyrosine kinases
SD: Stable disease
TACE: Transarterial chemoembolization
TKIs: Tyrosine kinase inhibitors
uHCC: Unresectable hepatocellular carcinoma
VEGFR: Vascular endothelial growth factor receptor
versus: vs.
